# Dietary diversity and associated factors among women attending antenatal clinics in the coast region of Tanzania

**DOI:** 10.1186/s40795-024-00825-1

**Published:** 2024-01-22

**Authors:** Rashidi Heri, Mats Malqvist, Khadija I. Yahya-Malima, Lilian Teddy Mselle

**Affiliations:** 1 Department of Nursing Management, Muhimbili University of Health and Allied Sciences, Dar es Salaam, Tanzania; 2 Department of Women’s and Children’s Health, Uppsala University, Uppsala, Sweden; 3 Department of Clinical Nursing, Muhimbili University of Health and Allied Sciences, Dar es Salaam, Tanzania

**Keywords:** Antenatal care, Dietary diversity, Nutrition knowledge, Pregnant women, Tanzania

## Abstract

**Background:**

Antenatal care (ANC) is crucial for reducing maternal morbidity and mortality, especially in low-resource settings. During antenatal care, women are provided with resources for enhancing their dietary diversity, like nutrition education and counseling. Improved nutrition knowledge influences positive nutritional behavior change, like women’s improved dietary diversity, which may increase the likelihood of a healthier pregnancy and delivery experience.

**Objective:**

This study aim was to assess dietary diversity and associated factors among pregnant women attending antenatal care in the Coast region of Tanzania.

**Methods:**

The descriptive cross-sectional study design was used to assess dietary diversity and associated factors among 338 pregnant women. A semi-structured questionnaire collected information from pregnant women on social demographic characteristics, nutrition knowledge and dietary diversity. Women were classified as having a varied diet if they had consumed at least five of the ten food groups over the previous twenty-four hours. Multivariable logistic regression analyses were used to identify predictors of dietary diversity in pregnant women.

**Results:**

Only 28% (95% CI: 23.5–33.1) (*n* = 95) of pregnant women met the minimum dietary diversity, and 18% (95% CI: 13.8–21.9) (*n* = 59) were considered to have a high level of nutrition knowledge. Living near a health facility (AOR = 1.77, CI 1.02, 3.06), having high nutrition knowledge (AOR = 2.58, 95% CI: 1.36, 4.89), and being pregnant for the first time (AOR = 2.44, 95% CI: 1.09, 5.44) were associated with adequate dietary diversity.

**Conclusion:**

Pregnant women in the study were found to have low knowledge about nutrition and inadequate dietary diversity intake. The findings underscore the need to improve nutrition knowledge provision in antenatal clinics by emphasizing the importance of a diversified and high-quality diet. Healthcare providers in antenatal care clinics should consistently provide nutrition education and counseling to pregnant women and promote their diversified food consumption. Such knowledge may eventually promote healthier pregnancy and child development by curbing the nutritional deficiencies experienced during pregnancy.

**Supplementary Information:**

The online version contains supplementary material available at 10.1186/s40795-024-00825-1.

## Introduction

Pregnancy is a critical period because of the increased demand for nutrition requirements due to the increased physiological and metabolic needs of the woman and the growing fetus [1]. To get all the required nutrients, women need to eat a variety of foods to enhance their nutritional needs. Dietary diversity may be defined as the consumption of a variety of food types over a determined period [2]. It is one of the components used to evaluate dietary quality and is an indicator of adequate nutrition. Micronutrient deficiency, also referred to as hidden hunger, is one of the problems affecting pregnant women worldwide. However, women in low-income countries are more vulnerable to deficiency due to inadequate and poor-quality diets [3]. Prenatal iron deficiency anemia is predicted to impact 15–20% of pregnant women worldwide, vitamin A deficiency affects 15% of pregnant women in low-income countries, and iodine deficiency is estimated to affect approximately 40% of pregnant women in Africa [3]. The prevalence of anemia among women of reproductive age has grown slightly from 31.6% in 2000 to 32.8% in 2016, although it has declined somewhat among pregnant women, from 41.6 to 40.0% over the same period [4]. Despite all efforts to alleviate such nutrition-related problems over time, they are still a challenge.

In Tanzania, anaemia affects 45% of women aged 15 to 49 on the Mainland and 60% in Zanzibar [5]. The Coast region ranks close to second in anaemia among women of childbearing age (15 to 59 years) at a rate of 58% and the highest being 71% in Mtwara while Dar es Salaam and Lindi are at 60% and 61% respectively. For children under two years, overall, 58% were anemic with hemoglobin levels of less than 11.0 g/dl [5] and we are uncertain of the current estimates. In the past decade, other nutritional-related disorders have also been on the rise. For example, in 2015, many women in Tanzania were overweight (Tanzania Demographic and Health Survey Report of 2015) [5], underscoring the need for nutritional behavioral change and the essence of improving nutritional literacy for pregnant women and the community.

Nutritional literacy is defined as “the degree to which individuals obtain, process, and understand nutrition information and skills needed to make appropriate nutrition decisions” [6]. To improve nutrition literacy, nutrition education is provided in antenatal clinics, where about 98% of Tanzanian pregnant women received antenatal care from skilled personnel at least once during their pregnancy [5]. The education is provided primarily by nurse midwives, who need to be trained to provide nutrition education in collaboration with nutritionists and dietitians, who are very few in the ANC settings in Tanzania. The nutrition education will equip pregnant women with knowledge that is aimed at improving their diet and nutrition status. However, the level of nutrition knowledge is still inadequate to influence behavioral change, so inadequate diet quality and diversity still prevail [7].

Inadequate nutrition knowledge for women is a prevailing problem in most developing countries including Africa. In Ethiopia, only 27% of 322 pregnant women reported having adequate nutrition knowledge [8] and in Kenya, 42% of 979 pregnant women had a nutrition knowledge score of 4.6 out of 11 [9]. A survey of 663 mothers and caregivers in rural households in Tanzania indicated that only 14% of them had received nutrition education before the survey [7]. Evidence from one RCT in rural Malawi revealed that pregnant women who participated in a community-based nutrition education intervention, had enhanced dietary diversity, increased nutrition knowledge, nutrition views, and behaviors, and changed their diets [10]. Also, a community-based nutrition education intervention has been found to improve the dietary and nutrition knowledge of caregivers, and the dietary diversity of children in Kenya and in Malawi has improved child dietary diversity even in a food insecure area [11, 12].

In Tanzania, little is known about dietary diversity practices, and associated factors among pregnant women. Few studies conducted on the topic, have focused mostly on the status of dietary diversity and indicated inadequate dietary diversity among women [13, 14]. Thus, it is important to assess the status of dietary diversity and associated factors, including nutrition knowledge, among antenatal clinic attendees to generate information for effective planning of nutritional education in ANC. This study was done in the coastal region, which has a higher prevalence of anemia among pregnant women and children. The study describes dietary diversity practices and associated factors among pregnant women in the Coast region of Tanzania.

## Materials and methods

### Study setting and design

This descriptive cross-sectional design was deployed using a nutritional knowledge and 24-hour dietary recall questionnaire to collect information on nutrition knowledge and dietary diversity among 338 pregnant women. Women between 20 and 26 weeks of gestation visiting the two health centers in Kibaha and Bagamoyo Districts of the Coast Region in March 2020 were eligible to participate in the study. This study was nested in a larger study that aimed to determine the effectiveness of a group antenatal care model on quality and satisfaction with antenatal care in semi-urban settings in Tanzania. The Kibaha and Bagamoyo District have a total population of 594,205 (290,202 males and 304,003 [15]. Specifically, the survey was conducted in three health centers and one district hospital: Mkoani and Mlandizi health centers in Kibaha District and Kerege and Bagamoyo district hospitals in Bagamoyo District. The three health centers and one district hospital were selected due to their high ANC attendance rates to enable reasonable time for allocating women to group antenatal care intervention.

### Sample size estimation and participants

The sample size was estimated by Epi Info 7 statistical software using a power of 80%, a two-sided confidence level of 95% and a ratio of unexposed and exposed of 1. The expected changes in women meeting adequate dietary diversity from 22 to 40% was estimated based on a study done in Kenya. The dropout rate of 15% was added to the sample size of 263 women. Women who attended the antenatal clinics at the Mkoani, Mlandizi, and Kerege health centers and the Bagamoyo district hospital were asked to take part in the study. To be involved in the study, a woman had to have a normal pregnancy and a gestational age of between 20 and 26. The gestational age restriction aimed to facilitate the early recruitment of eligible women for group antenatal care intervention, which requires pregnant women to be observed together throughout their pregnancy. In addition, to be recruited in group antenatal care intervention, women had to have not more than three ANC visits and less exposure to routine antenatal care and be able to speak and understand Kiswahili well. Four licensed midwives, one in each clinic were trained to serve as research assistants. Research assistants were trained on how to identify eligible participants during regular prenatal visits and how to administer the survey questionnaires. Women who agreed to take part in the study provided written informed consent.

### Data collection methods, and variables

#### Outcome variable

The outcome variable was women’s minimum dietary diversity (MDD-W). The dietary diversity score of pregnant women was measured using the Dietary Diversity for Women of Reproductive Age [16, 17].

#### 24-Hour dietary recall

A 24-hour dietary recall method was used to determine the dietary diversity of women. The women were asked to recount all foods and beverages they consumed during the 24 h between the time they woke up the previous day and the time they woke up on the day of the interview. The interviews were conducted at the ANC clinic in a quiet room out of reach of other people within the clinic’s vicinity. Women were also asked to estimate the portion sizes using household utensils and when and where the food was consumed.

#### Minimum dietary diversity for women (MDD-W)

Minimum Dietary Diversity for Women (MDD-W) was estimated using information collected from the 24-hour dietary recall [16]. Foods consumed in the past 24 h were grouped into one of the 10 food groups. These are “all starchy staples, beans and peas, nuts, and seeds, all dairy products, flesh foods (including organ meat and miscellaneous small animal protein like insects, insect larvae/grubs, insect eggs, and land and sea snails), eggs, vitamin A rich dark green leafy vegetables, other vitamin A-rich vegetables and fruits; other vegetables; and other fruits” [17]. Consumption of a particular food group scored 1, and if a group was not consumed a score of 0 was given. No minimum weight requirement was considered when classifying foods into food groups.

#### Predictor variables

Predictor variables included information on socio-demographic characteristics such as participants’ marital status, level of education, age, occupation, perceived income adequacy, number of pregnancies, having health insurance, pregnancy planning, perceived distance from a health facility, payment for an antenatal clinic, decisions for attending an antenatal clinic, anthropometric measures and pregnant women’s nutrition knowledge. The factors were explored using univariable analysis and all factors that achieved *p* ≤ 0.25 during univariable analysis [18] and those supported by the literature to be associated with dietary diversity were included in the multivariable logistic regression model. Perceived income adequacy and perceived distance from a health facility were defined and measured as follows: Perceived income adequacy refers to the manner in which a person or community subjectively evaluates their income to determine whether it is sufficient to meet their basic needs and maintain a certain standard of living [19]. To measure perceived income adequacy in this study, women were asked to rate how adequate their income was to meet their daily living expenses and were given the option to select whether their income was enough, barely enough, inadequate, or totally inadequate. Those who rated their income as enough were classified as having adequate income, and the rest were classified as having inadequate income.

Perceived distance from a health facility refers to an individual’s or a community’s subjective assessment of how far they perceive a healthcare facility to be. It is a subjective measure because it is based on people’s own perceptions and feelings about the distance from a heath facility [20]. To measure perceived distance from a health facility, women’s were asked to rate how far they live from their clinic or health facility. Participants were required to select given options including very near, near, far, and very far. Those who responded that they lived very near or near were classified as living near and those who said they lived far and very far were classified as living far.

#### Nutrition Knowledge

The nutrition knowledge questionnaire was used to determine pregnant women’s knowledge of food and nutrition. The questionnaire was composed of two sections: the maternal nutrition knowledge and food-related knowledge sections. The maternal nutrition knowledge section had 15 statements with 3 options, ‘true’, ‘false’, or ‘don’t know.” Each correct item in the maternal nutrition knowledge questionnaire was given a score of 1, while the false and don’t know items were given a score of 0 as shown in Table 2. Food-related knowledge section, which had 20 items, women were required to define a balanced diet, mention the benefits of a balanced diet and list food groups i.e. bodybuilding and blood-increasing foods, bone and tooth-enhancing foods, body-protecting foods (vitamin-rich foods), and energy-supplying foods. In this section, each correct item mentioned was given 1 point until the maximum score for each question was reached, as shown in Table 3. The overall questionnaire score was 35 points, and the higher the score, the greater the nutrition knowledge. The final maternal nutritional knowledge section with 15 items was tested for reliability and it had a high level of internal consistency with Cronbach’s alpha of 0.724. Additionally, nutritionists and nurse midwives reviewed the validity of the nutrition knowledge questionnaire. They reviewed the questionnaire and offered their feedback on how to make it better. Further, the pilot test was conducted before the main study to ensure the relevance and clarity of the questions, and their feedback was used to improve the questionnaire before data collection.

#### Anthropometric measures

Body weight was measured in kilograms to the nearest 0.1 kg using a Seca electronic adult scale with a stadiometer embedded for height measurement. To measure weight, women were required to remove their shoes and any bulky clothes. A single measurement was recorded to the nearest 0.1 kg [21]. The height of women was measured using a scale stadiometer with a sliding head plate and connecting rods marked with a measuring scale. Participants were asked to remove their shoes. The height of participants was taken while in the upright posture, standing before the stadiometer without shoes, with their shoulders, buttocks, and heels touching the measuring rod [22]. The reading was recorded to the nearest 0.5 cm. Measurements were taken by the licensed nurse midwives.

### Data analysis

The statistical package SPSS 26 was used to analyse the data. The normality of the distributions of all responses was determined numerically and visually. All variables were subjected to descriptive analysis. The score for maternal nutrition knowledge (15 items) was added to the food-related knowledge score (20 items) to get the overall nutrition knowledge score (35 items). The total of each subscale was divided by the number of items in the subscale to get an average score. The pregnant women’s knowledge scores for maternal nutrition knowledge and food-related knowledge were combined to get the overall nutrition knowledge. The average scores for maternal nutrition knowledge, food-related knowledge, and overall nutrition knowledge were divided by 15, 20, and 35, respectively, and converted to percentages by multiplying by 100. Those with a score of 80% or higher were considered to have high nutrition knowledge; those with a score between 60 and 79% were considered to have moderate nutrition knowledge; and those with a score of 0 to 59% were considered to have low nutrition knowledge [23]. Minimum Dietary Diversity for Women (MDD-W) was estimated and scored using information collected from the 24-hour dietary recall. Women with five or more food groups were considered to have high dietary diversity, while those with four or fewer food groups were considered to have low dietary diversity [16]. The binary logistic regression technique using enter variable selection was used to determine the factors that influenced dietary diversity. Those factors with *p* ≤ 0.25 in univariable analysis, were included in the logistic regression model [18] including those factors supported by literature to be associated with dietary diversity. Multicollinearity among the explanatory variables was checked using the variance inflation factor (VIF). VIF value ≤ 2.0 indicates the absence of multicollinearity. The significance level was set at 0.05.

### Ethics approval and consent to participate

The Muhimbili University of Health and Allied Sciences granted the study ethical clearance. All participants provided written consent after they told and understood the purpose and the procedure of the study, the voluntary nature of their participation, and the information they provided would remain confidential. All procedures were carried out following relevant guidelines and regulations.

## Results

### Socio-demographic characteristics of the pregnant women

The mean age of the women was 26.3 ± SD 5.9 years, their mean weight was 63.8 ± 12.4 kg, and their height was 156.3 ± 6.1 cm., 47.4% of the women, (*n* = 160), were under 156 cm in height, which is a small stature [24–26]. The mean gestational age in weeks for pregnant women was 23.12 ± SD 2.0 and most (99.7%, *n* = 337) were in their second trimesters. 88% (*n* = 290) of the participants were either married or cohabiting. When it came to education, 34.6% (*n* = 117) of participants had completed secondary education or more, while 56.5% (*n* = 191) of women had only finished primary school. Small businesses and agriculture were the main sources of income for 55.9% (*n* = 189) of women, and only 34.6% (*n* = 117) of women said their family’s income was enough to cover their requirements. 71% (*n* = 240) of the women said they lived far away from an ANC clinic. 31% of the women, (*n* = 105) were primigravida, and 32% (*n* = 105) of them had unplanned pregnancies (Table 1).

**Table 1:** Social demographic characteristics of pregnant women (*N* = 338)

Variables	n (%)
**Age in years** (***n* = 338)**	
≤ 24 Years	156 (45.6)
25–34 Years	144 (42.6)
≥ 35 Years	40 (11.8)
Mean (SD) Years	26.3(5.9)
**Gestation Age in Weeks**	
20–26 Weeks (2nd Trimester)	337(99.7)
27 Weeks (3rd Trimester)	1(0.3)
Mean (SD) Weeks	23.12(1.99)
**Height in Centimeters** (***n* = 338)**	
< 150 cm	29 (8.6)
150 to 155 cm	131(38.8)
156 to 160 cm	95 (28.1)
> 160 cm	83 (24.6)
Mean (SD) cm	156.3(6.1)
**Marital status** (***n* = 338)**	
Married	230 (68.0)
Single	40 (11.8)
Living together (Cohabiting)	68 (20.1)
**Level of Education** (***n* = 338)**	
No formal education	30 (8.9)
Primary education	191 (56.6)
Secondary education and higher	117 (34.6)
**Current occupation** (***n* = 338)**	
Wage employment	18 (5.3)
Business	173 (51.2)
Farming and Others	147 (43.5)
**Perceived income adequacy to meet family needs** (***n* = 338)**	
Enough	116 (34.3)
Inadequate	222 (65.7)
**Perceived distance from a health facility** (***n* = 338)**	
Near	98 (29.0)
Far	240 (71.0)
**Number of Pregnancy** (***n* = 338)**	
1	105 (31.1)
2 and 3	157 (46.4)
≥ 4	76 (22.5)
**Planned pregnancy** (***n* = 338)**	
Yes	230 (68)
No	108 (32)
**Having health insurance** (***n* = 338)**	
Yes	19 (5.6)
No	319 (94.4)
**Decisions for attending Antenatal Care Clinic**	
Myself	60 (17.8)
My husband, my partner, or others	30 (8.9)
Together with my husband or partner	248 (73.4)
**Payment for Antenatal Care Clinic** (*n*** = 338)**	
Myself	22 (6.5)
My husband, my partner, or others	316 (93.5)

### Maternal nutrition knowledge of pregnant women

Women were asked to respond to 15 maternal nutrition statements, and each correct response was given one point (Additional file 1). The women were supposed to answer yes, no, and I don’t know. The false answer and I don’t know were regarded as no knowledge and given a score of zero. The average maternal nutrition knowledge score was 9 ± SD 2.2. The average score percentage was 60.43 ± SD 14.3. When maternal nutrition knowledge was categorized, about two-thirds of pregnant women had moderate (48.8%, *n* = 165) to high (14.2%, *n* = 48) maternal nutrition knowledge, while the rest (37.0%, *n* = 125) had low maternal nutrition knowledge. Most pregnant women were aware of the significance of good food preparation hygiene, balanced nutrition from a variety of foods, regular exercise during pregnancy, plenty of water consumption during pregnancy, increased food intake during breastfeeding, and the role partners play in improving pregnant women’s nutrition and health. Additional information is found in Table 2.

**Table 2:** Maternal nutrition knowledge of pregnant women (*n* = 338)

Sn	Maternal Nutrition Knowledge (15 Items)	A umber of women with correct Answers n(%)	A number of women with False Answers n(%)	A number of women who don’t know the answers. n(%)
1	The fetus is most vulnerable to nutrition deficiencies in the first trimester of pregnancy	229 (67.8)	26 (7.7)	83 (24.6)
2	Women who are overweight or obese are allowed to lose weight during pregnancy	129 (38.2)	90 (26.6)	119 (35.2)
3	To gain weight during pregnancy women is not necessary	176 (52.1)	106 (31.4)	56 (16.6)
4	Unhygienic food preparation may lead to diseases, poor fetus growth, and miscarriage	315 (93.2)	10 (3.0)	13 (3.8)
5	It is advised to avoid eating food with teas, coffee and soda because they may lead to anemia	160 (47.3)	79 (23.4)	99 (29.3)
6	It is advised balanced diet to be based on mixed type of food groups	311 (92.0)	10 (3.0)	17 (5.0)
7	A pregnant woman with normal weight is anticipated to gain of 6–9 kg during pregnancy	33 (9.8)	142 (42.0)	163 (48.2)
8	Underweight during pregnancy do not have any affect to the delivered baby	141 (41.7)	92 (27.2)	105 (31.1)
9	Pregnant women are not required to exercise	289 (85.5)	43 (12.7)	6 (1.8)
10	To drink plenty of water is not important to pregnant women	286 (84.6	48 (14.2)	4 (1.2)
11	An HIV infection does not increase energy and nutrient needs.	146 (43.2)	74 (21.9)	118 (34.9)
12	A woman who is malnourished can still adequately breastfeed her baby	28 (8.3)	214 (63.3)	96 (28.4)
13	Men can help improve women’s nutrition by helping them with their workload.	281 (83.1)	33 (9.8)	24 (7.1)
14	Breastfeeding mothers should eat more than women who is not breastfeeding	324 (89.6)	23 (6.8)	12 (3.6)
15	Iodized salt is important in brain and nervous system development	237 (70.1)	14 (4.1)	87 (25.7)

### Food-related knowledge of pregnant women

Women were asked to define a balanced diet, mention the benefits of a balanced diet, and list food groups, bodybuilding and blood-increasing foods, bone and tooth-enhancing foods, body-protecting foods (vitamin-rich foods), and energy-supplying foods. Each correct item was given one point until a maximum of 20 was reached, as shown in Table 3 below.

The average food-related knowledge score obtained was 7.51 ± 3.14. The average percentage score was 37.58 ± 15.74, and when the women food-related knowledge was categorized, the majority had low food-related knowledge, (89.1%, *n* = 301), the rest had moderate food-related knowledge (9.5%, *n* = 32), and high food-related knowledge (1.5%, *n* = 5).

**Table 3:** Food-related knowledge of pregnant women (*n* = 338)

Number of items scored	Food Related Knowledge (20 Scored Items)		*n* = 338	Percent
1	Balanced Diet Definition	A diet based on mixed types of food groups that provide all nutrients required by the body	130	38.5
		A diet that provides all nutrients required by the body	52	15.4
2	Balanced Diet Benefit	Provide energy and heat to the body	194	57.4
		Helps the health of the mother and the growth of the fetus	221	65.4
		Protect the body	87	25.7
		Helps the body to function properly	46	13.6
4	Food groups	Cereals, tubers, and banana	150	74.0
		Pulses and food of animal origin	145	42.9
		Fruits	113	33.4
		Vegetables	143	42.3
		Milk and milk products	44	13.0
4	Bodybuilding and Blood increasing food	Green vegetables	318	94.1
		Liver	18	5.3
		Beans	133	39.3
		Meat	35	10.4
		Eggs	13	3.8
		Fruits	78	23.1
3	Bone and teeth-enhancing foods that	Milk and milk products	11	3.3
		Fish consumed whole (Sardine etc.)	87	25.7
		Green vegetables	22	6.5
2	Body Protecting foods (Vitamins rich food)	Green and yellow vegetables	95	28.1
		Fruits	81	24.0
4	Energy Providing Food	Cereals, tubers, and banana	192	56.8
		Nuts	24	7.1
		Sweet fruits	38	11.2
		Sugar and honey	13	3.8
		Fat and oil	27	8.0
Total 20				

### Dietary diversity of pregnant women

Minimum Dietary Diversity for Women (MDD-W) were estimated using information collected from the 24-hour dietary recall [16, 17], based on the list of food consumed in each group (Additional file 2). If a food was consumed once or more times in the previous 24 h, it was deemed to be part of the food group. Foods consumed in the past 24 h were grouped into one of the 10 food groups. The women who consumed 5 or more food groups are regarded as achieved minimum dietary diversity for women while those with four groups or low did not meet the minimum dietary diversity. Most of the women in this study reported consuming their food at home (96.4%, *n* = 326) and the rest consumed their food at their place of work and home (2.1%, *n* = 7), restaurant and home (0.9%, *n* = 3) and home and on the way (0.6%, *n* = 2). The average dietary diversity obtained was 3.89 ± 1.18. The average percentage score was 38.90 ± 11.84, and when the dietary diversity was categorized, the majority had not met minimum dietary diversity, (72.8%, *n* = 246), and only (27.2%, *n* = 92) met minimum dietary diversity. All women consumed all of the starches in the staple food group, followed by dark-green vegetables and flesh foods. The least consumed food groups were eggs, nuts and seeds, and all dairy products (Table 4).

**Table 4:** Dietary diversity among pregnant women attending ANC in coast region, Tanzania (*n* = 338)

Food Group	*n* = 338	Percent
All starchy staples	338	100.0
Beans and peas	132	47.9
Nuts and seeds	16	4.7
All dairy	47	13.9
Flesh foods (including organ meat and miscellaneous small animal protein (insects, insect larvae/grubs, insect eggs, and land and sea snails)	206	60.9
Eggs	15	4.4
Vitamin A rich dark green leafy vegetables	216	63.9
Other vitamin A rich vegetables and fruits^2^	102	30.2
Other vegetables	73	21.6
Other fruits	140	41.4

### Factors affecting pregnant women dietary diversity

The univariable logistic regression analysis was done to explore the unadjusted association of a range of factors with pregnant women’s dietary diversity. Three of the independent variables (number of pregnancies, pregnant women’s nutrition knowledge, and distance from a health facility) were found to predict the variety of foods pregnant women eat. The odds of having adequate dietary diversity were 2.44 times (AOR = 2.44, 95% CI: 1.09, 5.44) higher among pregnant women who were in their first pregnancies than those who were in their fourth or more pregnancies. Also, the odds of having adequate dietary diversity were 2.58 (AOR = 2.58, CI 1.36, 4.89) higher in pregnant women who had high nutrition knowledge than in those with low nutrition knowledge. Also, the odds of having adequate dietary diversity were 1.77 (AOR = 1.77, CI 1.02, 3.06) higher in pregnant women who lived near an antenatal clinic than in those who lived far from the clinic (Table 5).

**Table 5:** Factor associated with dietary diversity among pregnant women attending ANC in coastal region, Tanzania (*n* = 338)

Variable	Dietary Diversity level		COR (95%CI)	AOR (95%CI)
	Adequate (n)	Inadequate (n)		
**Marital status**	60	170		
Married	15	25	1	1
Single	17	51	1.70 (0.84, 3.43)	1.33 (0.61, 2.89)
Living together (Cohabiting)			0.94 (0.50, 1.76)	0.92 (0.47, 1.80)
**Level of Education**				
No formal education and primary School education	61	160	1.05 (0.63, 1.75)	1.36 (0.77, 2.40)
Secondary education and higher	31	86	1	1
**Current occupation**				
Homemakers/Housewives	63	144	1.53 (0.93, 2.55)	1.51 (0.87, 2.62)
Working/Have some income	29	102	1	1
**Having health insurance**				
Yes	9	10	2.55 (1.00, 6.51)*	2.20 (0.81, 6.00)
No	83	236	1	1
**Number of Pregnancy**				
1	32	73	1.64 (0.82, 3.27)	2.44 (1.09, 5.44)*
2 and 3	44	113	1.46 (0.76, 2.80)	1.82 (0.89, 3.70)
≥ 4	16	60	1	1
**Decisions for attending Antenatal Care Clinic**				
Myself, my husband, my partner, or others	32	58	1.72 (1.02, 2.90)*	1.48 (0.81, 2.69)
Together with my husband or partner	60	188	1	1
**Nutrition Knowledge**				
Medium to High Knowledge	25	34	2.32 (1.29, 4.17)*	2.58 (1.36, 4.89)*
Low Knowledge	67	212	1	1
**Distance from health facility**				
Near			0.52 (0.31, 0.87)*	1.77 (1.02, 3.06)*
Far			1	1

NB: Reference group; COR: crude odds ratio; AOR: adjusted odds ratio

^∗^statistically significant at P value < 0.05

## Discussion

Three hundred and thirty-eighty pregnant women with a mean age of 26 years and a mean gestation age of 23.1 weeks attending antenatal clinics were assessed for their dietary diversity practices and their associated factors, including their level of nutrition knowledge in the Coast region of Tanzania. The findings revealed that pregnant women had low levels of nutrition knowledge and dietary diversity practices. Nutrition knowledge is an important factor in determining dietary behavior including selecting quality food and ensuring adequate dietary diversity. High nutrition knowledge was found to be one of the important modifiable predictors of dietary diversity. Nutrition education enhances pregnant women’s nutrition knowledge and therefore increases their ability to select and prepare a diet with appropriate diversity and quality for the betterment of their health and that of their growing fetus [10, 27]. Nutrition knowledge has also been found to predict dietary diversity in Malawi [10] and Nepal [27] however, a study in Ghana suggests otherwise [28]. The reason for the difference could be due to the study setting, participant differences, or the method of measuring nutrition knowledge. Even though there is some disagreement, antenatal clinics should keep working to improve how they teach pregnant women about nutrition. This will help women learn more about nutrition, which will lead to improvement in their nutrition knowledge, dietary diversity, and health.

Nutrition education in schools and communities may improve people’s dietary diversity, especially among youth and women of reproductive age. In the Tanzanian school system, we have primary and secondary schools. In all primary schools where the majority of Tanzanians manage to attend, the content of food and nutrition is learned in science subjects, in which there are a few sub-topics, including health care and environment, whereby students learn about food groups and a few nutritional disorders. For those who manage to go to secondary schools, food, and nutrition subjects are taught in a few selected secondary schools at the ordinary level of education and in even fewer schools at the advanced secondary school level. In the majority of secondary schools, nutrition is covered as a topic in the biology subject in one topic of food nutrients and their disorders [29, 30]. Community-based nutrition education interventions were reported to improve the dietary and nutrition knowledge of caregivers and children’s dietary diversity in Kenya [11]. In addition, a cluster randomized controlled trial using participatory community-based nutrition education for caregivers done in Malawi has improved child dietary diversity even in a food-insecure area [12]. Nutrition education interventions are therefore important in improving nutrition knowledge and dietary diversity in the community and may also work in a hospital setting.

In Tanzania, almost all pregnant women (98%) receive skilled antenatal care at least once during pregnancy and more than half (51%) have four or more antenatal visits [5]. During these visits, pregnant women receive nutrition education and other nutrition services, like iron and folic acid supplementation, weight measurements, dietary assessments, and hemoglobin level monitoring [31, 32]. Despite this effort, only about one-fifth of women (17.5%) had high overall nutrition knowledge, with women knowing more about maternal nutrition (66%) than food-related knowledge (11%). This may be because nutrition information is given in a general manner in such a way that most women know general nutrition concepts like the importance of hygienic food preparation, a balanced diet, regular exercise, and drinking plenty of water during pregnancy, but they don’t know the details and the functions of different food groups and their contribution to maternal nutrition. Also, only 9.8% of women know the appropriate weight gain needed during pregnancy, and this may impair their ability to monitor their health during pregnancy by increasing food intake, especially involving a variety of foods. Low nutrition knowledge was also found among mothers and caretakers in the community, which reflects the need to extend nutrition education to the community [7].

Diversified food enables women to get all the required nutrients and it is known to improve maternal and newborn outcomes including improving health during pregnancy and lowering the risk of anaemia during pregnancy and the risk of having a baby with low birth weight [1, 33, 34]. Also, it has been found to reduce the risk of death in a systematic review by Mozaffari (2022), which found a 22% reduced risk of death in adults consuming diets with four or more food groups [35]. In Tanzania, however, the higher Dietary Diversity Score (DDS) was associated with a lower risk of small for gestational age (SGA) [36]. Therefore, interventions to improve dietary diversity are important to improve the health of the population, especially for pregnant women and children.

This study indicated that most women’s diets consisted of cereals, roots and tubers, vegetables, legumes, nuts and seeds, and fruit, similar to other studies done in Tanzania [13, 14]. The dietary diversity practice among pregnant women was low, and only about one-third of pregnant women (27.2%) met the minimum dietary diversity standard; other studies conducted in Tanzania indicated slightly higher dietary diversity, where about half of the women met minimum dietary practices [13, 14]. The reasons may be the seasonality, and the setting, where two of those studies involved pregnant women in the antenatal clinic, and one involved rural family household. The one with the lowest practice was done in semi-urban areas, followed by the one involving rural family households, and the last in the rural ANC clinic. Rural area women may also have had more access to food from farming and eating at low cost, and this may have contributed to a slightly higher diversity compared to those from semi-urban areas. Low nutrition education among the general population [7] may have also contributed to low dietary diversity, for in our setting, very few people have undergone nutrition education, which is one of the important factors associated with low dietary diversity in women.

Low dietary diversity is a widespread problem among women, especially in developing countries [37–39]. Systematic reviews and meta-analyses done in Ethiopia have shown a high prevalence of inadequate dietary diversity among pregnant women at the national level and dietary practices of women that were suboptimal and below WHO and FAO recommendations [40, 41]. Also, the determinants of dietary diversity practice were, mothers who can read and write; maternal primary school and above education; nutritional information; dietary diversity knowledge; and households with a high wealth index [42]. Pregnant women need to be better nourished, and more needs to be done to help them. So, efforts should be made to improve nutrition education provisions in the community and the ANC clinic to increase women’s dietary diversity.

Predictors of pregnant women’s dietary diversity were the level of nutrition knowledge, number of pregnancies, and distance from a health facility. Women with high nutrition knowledge were more than twice as likely to achieve adequate dietary diversity compared to those with low nutrition knowledge. This may be because the women know the importance of a balanced diet during pregnancy and the need for dietary diversity. Also, high nutrition knowledge was found to be a predictor of adequate dietary diversity in studies done in Ethiopia [1, 43]. Even though having more pregnancies predicted the odds of having high nutrition knowledge, it did not predict adequate nutrition diversity, with first-time mothers being more likely to achieve adequate dietary diversity compared to those with a second pregnancy and above. This could be because health workers, family members, and friends pay more attention to and give advice to first-time pregnant women about what to eat. These women may then follow this advice because they want to take care of their first baby and feel pressured to do so. Distance from the health facility was also found to predict dietary diversity adequacy, indicating that women who lived close to their health facility were more likely to meet adequate dietary diversity than those who lived far away. This may be caused by the women living nearby arriving early in the clinic without much hassle, which may lead to tiredness and poor attention to the education session, especially for those women from a far distance. Even though nutrition knowledge is a major predictor of dietary diversity, other factors, such as the number of pregnancies and distance from a health facility, may play a role. It is important to advise experienced women to continue to follow nutrition advice by including a variety of foods in their diet and not to do business as usual.

### Limitations of study

Social desire bias may be one of the study’s limitations. However, every effort was made to reduce bias by providing intensive training for data collectors on how to probe women to recall their dietary practices.

## Conclusions and recommendations

The Overall dietary diversity level was low among pregnant women in the study area. Also, nutritional knowledge levels were low among pregnant women. Dietary diversity was associated with the level of nutrition knowledge, number of pregnancies, and distance from a health facility.

Effort from all sectors is needed to improve women’s nutrition literacy, nutrition education, and counseling in our antenatal setting, including advocating for dietary diversity for pregnant women, partner involvement in antennal care services, and making sure antenatal care is provided as near as possible to pregnant women, including follow-up in the community. Also, the importance of dietary diversity needs to be emphasized during nutrition education and counseling sessions tailored to each woman’s needs and risks so that all women achieve the required dietary diversity and improve their health and that of their fetus. Also, there is a need to investigate the behavioral change strategies related to healthy food choices and explore the effectiveness of education programs on women’s dietary diversity.

### Electronic supplementary material

Below is the link to the electronic supplementary material.

**Supplementary Material 1:** Pregnant women’s maternal nutrition knowledge tool answer keys.

**Supplementary Material 2:** Pregnant women’s maternal nutrition knowledge tool answer keys description

**Supplementary Material 3:** List of food consumed in each group of the minimum dietary diversity for women (MDD-W)

**Supplementary Material 4:** List of food consumed in each group of the minimum dietary diversity for women (MDD-W) description

## Data Availability

The datasets used and/or analysed during the current study are available from the corresponding author upon reasonable request.

## Abbreviations

ANC: Antenatal Care
MDD-W: Minimum Dietary Diversity for Women
DDS: Dietary Diversity Score
TDHS: Tanzania Demographic and Health Survey
MoHCDEC: Ministry of Health, Community Development, Gender, Elderly and Children
URT: The United Republic of Tanzania
WHO: World Health Organization
FAO: Food and Agriculture Organization of the United Nations
