# Supplementary material for: Dietary diversity and associated factors among women attending antenatal clinics in the coast region of Tanzania
Source: BMC Nutr. 2024 Jan 22;10:16. doi: 10.1186/s40795-024-00825-1 (PMC10801968; doi:10.1186/s40795-024-00825-1)
Supplement: Supplementary file 1 — Supplementary Material 1: Pregnant women’s maternal nutrition knowledge tool answer keys. [file 40795_2024_825_MOESM1_ESM.docx]

**Pregnant Women Maternal Nutrition Knowledge Answer Key**

| **Sn** | **Maternal Nutrition Knowledge (15 Items)** | |
| --- | --- | --- |
| **1** | **The fetus is most vulnerable to nutrition deficiencies in the first trimester of pregnancy** | **TRUE** |
| **2** | **Women who are overweight or obese are allowed to lose weight during pregnancy** | **FALSE** |
| **3** | **To gain weight during pregnancy women is not necessary** | **FALSE** |
| **4** | **Unhygienic food preparation may lead to diseases, poor fetus growth, and miscarriage** | **TRUE** |
| **5** | **It is advised to avoid eating food with teas, coffee and soda because they may lead to anemia** | **TRUE** |
| **6** | **It is advised balanced diet to be based on mixed type of food groups** | **TRUE** |
| **7** | **A pregnant woman with normal weight is anticipated to gain of 6–9 kilograms during pregnancy** | **FALSE** |
| **8** | **Underweight during pregnancy do not have any affect to the delivered baby** | **FALSE** |
| **9** | **Pregnant women are not required to exercise** | **FALSE** |
| **10** | **To drink plenty of water is not important to pregnant women** | **FALSE** |
| **11** | **An HIV infection does not increase energy and nutrient needs.** | **FALSE** |
| **12** | **A woman who is malnourished can still adequately breastfeed her baby** | **TRUE** |
| **13** | **Men can help improve women's nutrition by helping them with their workload.** | **TRUE** |
| **14** | **Breastfeeding mothers should eat more than women who is not breastfeeding** | **TRUE** |
| **15** | **Iodized salt is important in brain and nervous system development** | **TRUE** |
