# Supplementary material for: Dietary diversity and associated factors among women attending antenatal clinics in the coast region of Tanzania
Source: BMC Nutr. 2024 Jan 22;10:16. doi: 10.1186/s40795-024-00825-1 (PMC10801968; doi:10.1186/s40795-024-00825-1)
Supplement: Supplementary file 2 — Supplementary Material 2: Pregnant women’s maternal nutrition knowledge tool answer keys description [file 40795_2024_825_MOESM2_ESM.docx]

**Pregnant Women's Maternal Nutrition Knowledge Tool Answer Keys**

Additional File 1 contains the answer key for the pregnant women's maternal nutrition knowledge tool. True means the statement is true, and false means the statement is not true.
