# Supplementary material for: Dietary diversity and associated factors among women attending antenatal clinics in the coast region of Tanzania
Source: BMC Nutr. 2024 Jan 22;10:16. doi: 10.1186/s40795-024-00825-1 (PMC10801968; doi:10.1186/s40795-024-00825-1)
Supplement: Supplementary file 3 — Supplementary Material 3: List of food consumed in each group of the minimum dietary diversity for women (MDD-W) [file 40795_2024_825_MOESM3_ESM.docx]

**List of Food Consumed in Each Group of the Minimum Dietary Diversity for Women (MDD-W)**

|  | **Food Group** | **Food consumed** |
| --- | --- | --- |
| 1 | All starchy staples | **Maize/Corn, rice, wheat, sorghum, millet, cassava, banana, white potatoes and food made from these, ie. Stiff porridge (Ugali), rice, porridge, pasta, spaghetti.** |
| 2 | Beans and peas | **Beans, peas, cowpea, lentils** |
| 3 | Nuts and seeds | **Peanuts and Cashew nuts** |
| 4 | All dairy | **Milk and yogurt** |
| 5 | Flesh foods (including organ meat and miscellaneous small animal protein) | **Cow meat, fish, sardine, organ meat** |
| **6** | **Eggs** | **Chicken eggs** |
| 7 | Vitamin A rich dark green leafy vegetables | **Sweet potatoes leaves (matembele), Amaranth (mchicha), Cassava leaves (kisamvu), Pumpkin leaves (Msusa), Cabbage, Chinese, Ocra leaves, green sweet pepper, spinach** |
| 8 | Other vitamin A rich vegetables and fruits^2^ | **Pumpkins, carrot, yellow sweet potatoes, ripen mangoes and ripen papaya, red sweet pepper** |
| 9 | Other vegetables | **Tomatoes, onions, eggplant, ocra, cabbage, mushrooms, white eggplant (nyanya chungu)** |
| 10 | Other fruits | **Guava, Passion fruit, jackfruit, tamarind, watermelon, ripen banana, pineapple, apple.** |
