# Supplementary material for: Dietary diversity and associated factors among women attending antenatal clinics in the coast region of Tanzania
Source: BMC Nutr. 2024 Jan 22;10:16. doi: 10.1186/s40795-024-00825-1 (PMC10801968; doi:10.1186/s40795-024-00825-1)
Supplement: Supplementary file 4 — Supplementary Material 4: List of food consumed in each group of the minimum dietary diversity for women (MDD-W) description [file 40795_2024_825_MOESM4_ESM.docx]

**List of Food Consumed in Each Food Group of Minimum Dietary Diversity for Women (MDD-W)**

Additional File 2, contains a list of foods consumed by pregnant women mentioned during the 24-hour diet recall and summarized by 10 groups of the Minimum Dietary Diversity for Women (MDD-W).
